# Unravelling Antimicrobial Resistance in *Mycoplasma hyopneumoniae*: Genetic Mechanisms and Future Directions

**DOI:** 10.3390/vetsci11110542

**Published:** 2024-11-05

**Authors:** Raziallah Jafari Jozani, Mauida F. Hasoon Al Khallawi, Darren Trott, Kiro Petrovski, Wai Yee Low, Farhid Hemmatzadeh

**Affiliations:** 1 Australian Centre for Antimicrobial Resistance Ecology, Faculty of Sciences, Engineering and Technology, School of Animal and Veterinary Science, The University of Adelaide, Adelaide, SA 5005, Australia; mauida.alkhallawi@adelaide.edu.au (M.F.H.A.K.); darren.trott@adelaide.edu.au (D.T.); kiro.petrovski@adelaide.edu.au (K.P.); farhid.hemmatzadeh@adelaide.edu.au (F.H.); 2 The Davies Livestock Research Centre, School of Animal and Veterinary Sciences, The University of Adelaide, Adelaide, SA 5005, Australia; wai.low@adelaide.edu.au

**Keywords:** *Mycoplasma hyopneumoniae*, antimicrobial resistance, pigs, enzootic pneumonia, resistance mechanisms

## Abstract

Antimicrobial resistance (AMR) in *Mycoplasma hyopneumoniae*, the causative agent of Enzootic Pneumonia in swine, poses a significant challenge to the swine industry. This review focuses on the genetic foundations of AMR in *M. hyopneumoniae*, highlighting the complexity of resistance mechanisms, including mutations, horizontal gene transfer, and adaptive evolutionary processes. Techniques such as Whole Genome Sequencing (WGS) and multiple-locus variable number tandem repeats analysis (MLVA) have provided insights into the genetic diversity and resistance mechanisms of *M. hyopneumoniae*. The study underscores the role of selective pressures from antimicrobial use in driving genomic variations that enhance resistance. Additionally, bioinformatic tools utilizing machine learning algorithms, such as CARD and PATRIC, can predict resistance traits, with PATRIC predicting 7 to 12 AMR genes and CARD predicting 0 to 3 AMR genes in 24 whole genome sequences available on NCBI. The review advocates for a multidisciplinary approach integrating genomic, phenotypic, and bioinformatics data to combat AMR effectively. It also elaborates on the need for refining genotyping methods, enhancing resistance prediction accuracy, and developing standardized antimicrobial susceptibility testing procedures specific to *M. hyopneumoniae* as a fastidious microorganism. By leveraging contemporary genomic technologies and bioinformatics resources, the scientific community can better manage AMR in *M. hyopneumoniae*, ultimately safeguarding animal health and agricultural productivity. This comprehensive understanding of AMR mechanisms will be beneficial in the adaptation of more effective treatment and management strategies for Enzootic Pneumonia in swine.

## Simple Summary

Antimicrobial resistance (AMR) in bacteria is a critical issue threatening both human and animal health. This paper focuses on *Mycoplasma hyopneumoniae*, a bacterium causing lung disease in pigs, leading to significant economic losses in the swine industry worldwide. The problem is that this bacterium has developed resistance to many antibiotics, making treatment difficult. The study aims to understand the genetic basis of AMR by analyzing the reported genome of *Mycoplasma hyopneumoniae* strains using advanced techniques like whole genome sequencing. Key findings indicate that genetic mutations in certain genes are responsible for this resistance. This review paper suggests a multidisciplinary approach combining genetic, phenotypic, and bioinformatics data is essential in combating ever-increasing AMR in *Mycoplasma hyopneumoniae*. These insights could lead to better treatment strategies, ultimately benefiting the swine industry by improving animal health and reducing economic losses. Understanding and managing AMR in *Mycoplasma hyopneumoniae* is crucial for developing more effective antimicrobial agents and securing sustainable food production, which has a direct impact on society by ensuring food security and animal welfare.

## 1. Introduction

Antimicrobial resistance (AMR) has become one of the largest challenges in modern health, posing threats to both human and animal populations. While antimicrobial agents once seemed to be the solution to infectious diseases, today’s reality is quite different, with almost all pathogenic bacteria showing resistance traits against antimicrobial agents [1]. This problem not only strains human health but also threatens sustainable food production and environmental health, requiring coordinated global efforts to address it [2]. In response, the Food and Agriculture Organization (FAO), the World Organization for Animal Health (WOAH), and the World Health Organization (WHO) have announced a global action plan to combat AMR [3]. This plan emphasizes the standardization of surveillance methods for AMR across nations, in line with the provisions of the Terrestrial Animal Health Code, and encourages countries to implement domestic action plans to monitor and assess AMR [4]. Although AMR-associated foodborne and zoonotic bacterial pathogens are known to transfer between animals and humans, it remains unclear if animal antimicrobial use directly drives AMR in humans [5]. However, there is a wide consensus that the use of antimicrobials in farm animals—whether as therapeutic agents or growth promoters—contributes to the global AMR challenge [6,7].

*Mycoplasma hyopneumoniae* (*M. hyopneumoniae*), the causative agent of Enzootic Pneumonia (EP) in pigs, presents a significant concern within the swine industry due to its persistent presence and the economic losses it causes [2]. First, isolated in the 1930s and identified as the causative agent in the 1960s, *M. hyopneumoniae* remains a difficult organism about which research and control is hard to conduct [4]. The bacteria cause respiratory infections and predispose pigs to secondary bacterial infections, complicating welfare issues and production [1]. Though various antimicrobials are employed for the control of EP, the emergence of resistance in *M. hyopneumoniae* strains has been reported by researchers from different parts of the world, raising concerns about the efficacy of current treatments and underscoring the need for deeper genetic and phenotypic investigations into this pathogen [5].

AMR in *M. hyopneumoniae* involves complex genetic mechanisms, including mutations, horizontal gene transfer, and adaptive evolutionary processes [8]. Recent studies have emphasized the role of genome-wide approaches for unraveling resistance traits in the compact genome of *M. hyopneumoniae*. Methods like Whole Genome Sequencing (WGS), multiple-locus variable number tandem repeats analysis (MLVA), and other techniques of genotyping help us understand the genetic diversity and resistance mechanisms of *M. hyopneumoniae* [1,2]. These studies now illustrate how selective pressures exerted by the mass use of antimicrobials can drive genomic variations that increase AMR, thus challenging effective treatment and control of the disease [8]. The term AMR refers to bacteria gaining new genes or developing mutations in their genomic DNA, which helps them survive and grow even when exposed to antimicrobial drugs [2]. Bacteria may also exhibit behaviors that protect them against antimicrobials, such as forming biofilm structures, which show higher antibiotic resistance compared to free-living bacteria [6]. Bioinformatics can help combine genetic data with phenotypic AMR profiles to make much better predictions of potential resistance traits [3]. Databases, such as the Comprehensive Antibiotic Resistance Database (CARD) and the Pathosystems Resource Integration Center (PATRIC), therefore, provide very useful facilities for researchers. These databases provide an aggregation of AMR genes and related mechanisms, thereby allowing the capability of precise identification and annotation of resistance determinants [9]. The utilization of machine learning algorithms in these platforms further enhances prediction capabilities, enabling researchers to anticipate resistance phenotypes and their associated genomic attributes, thus providing a more proactive approach to managing AMR [2,3].

Mitigation of AMR in *M. hyopneumoniae* relies on an integrated multidisciplinary genomics approach combined with phenotyping and bioinformatics. Future research should focus on optimizing the genotyping methods, such as long-read sequencing technologies, to improve resistance predictions and develop standardized antimicrobial susceptibility testing procedures specific to *M. hyopneumoniae*. Additionally, there are evolutionary pressures and genetic adaptations to resistance that can be taken into consideration in the development of more effective antimicrobial strategies [4]. Instead, modern genomic technologies and bioinformatic tools can be applied by scientists to help them better combat the increasing threat of AMR in *M. hyopneumoniae* and other microorganisms, ultimately safeguarding animal health and agricultural productivity [2].

This paper will provide an explanation of the genetic basis for AMR in *M. hyopneumoniae*, considering recent progress made in genomics and bioinformatics. It seeks to provide a comprehensive understanding of the resistance mechanisms at play within this pathogen. The article will discuss the identification and characterization of resistance genes, analyze the implications of genetic diversity on AMR, and evaluate how these findings can inform better treatment and management strategies for combating EP in the swine industry [3]. Through this investigation, we aim to contribute valuable insights to the ongoing efforts to mitigate the detrimental impact of AMR in the veterinary and agricultural realms.

## 2. *Mycoplasma hyopneumoniae*: The Etiologic Agent of Enzootic Pneumonia (EP)

While the first written account of EP traces back to 1933 [7], concrete evidence of its causal agent did not emerge until 1963. In the 1950s, certain facets of *M. hyopneumoniae* captured researchers’ attention; it emerged as a fastidious organism, and during growth on solid media, *M. hyorhinis* typically outpaced *M. hyopneumoniae* [8]. Amidst some discrepancies among researchers from the UK and the US, the designation “*Mycoplasma hyopneumoniae*” was eventually attributed to the source of enzootic pneumonia (EP). Respiratory infections caused by *M. hyopneumoniae* in pigs rank among the foremost concerns in the swine industry. These infections persist subtly within substantial pig populations for an uncertain and often extended duration, yielding significant economic repercussions during the post-weaning period. Notably, they not only render pigs susceptible to additional bacterial infections but also give rise to intricate welfare complications. Within the field of veterinary medicine, employing antimicrobials to combat Enzootic Pneumonia (EP) is a prevalent practice. Veterinarians, guided by the pharmacopeia of their respective countries, prescribe single or combined antimicrobial agents to combat *M. hyopneumoniae* [9]. The present review seeks to deepen the comprehension of existing research that investigates the genetic underpinnings of *M. hyopneumoniae* and its association with the AMR trait as the causative agent of EP in pigs.

Regarding classification, *M. hyopneumoniae* finds its place within the Class Mollicutes, the order Mycoplasmatales, and the family Mycoplasmataceae. A process of reductive evolution has guided its evolutionary course away from the cell wall-bearing Gram-positive ancestors, such as *Clostridium*, and allowed it to become the smallest free-living organism devoid of a well-defined cell wall. Through a DNA sequencing analysis of the 16S rRNA, *M. hyopneumoniae*, *M. hyorhinis*, and *M. flocculare* form a phylogenetic cluster termed Neurolyticum. Recent genome sequence-driven taxonomic studies, however, have assigned *M. hyopneumoniae* to a novel genus called *Mesomycoplasma* [10].

In terms of its genome, *M. hyopneumoniae’*s distinctive characteristics include its modest genome size (~0.9 kb), GC content of ~30%, the conversion of the UAG stop codon to tryptophan during translation, and low GC content. These attributes strongly suggest the presence of precise transcription terminators [11], illuminating why its genome structure lacks intermittent non-functional DNA and pseudogenes. This genome adaptation appears tailored to meeting minimal life requisites [9].

## 3. Genome-Wide Studies and Antimicrobial Resistance

### 3.1. Challenges and Limitations

Until now, genome-wide studies on *M. hyopneumoniae* remain limited as there are only 24 assembled and annotated genomes (https://www.ncbi.nlm.nih.gov/datasets/genome/?taxon=2099 (accessed on 20 October 2022) available on NCBI and a significant portion of these studies do not delve into the AMR traits of the bacterium directly. Employing pan-genomic approaches to unveil and expose concealed elements of AMR within *M. hyopneumoniae’*s compact genome is imperative. This effort is crucial for obtaining a more comprehensive and anticipatory perspective of strains that challenge the effectiveness of antimicrobial treatments.

In addition to factors like colonization in novel environments, the potential selective pressures of antimicrobials prompt certain strains to exhibit genomic variations. This can occur through mechanisms such as point mutations, homologous recombination, and horizontal gene transfer [12,13]. This genetic diversity leads to a higher level of resistance compared to non-treated isolates, which are still vulnerable to the same antimicrobial agents [14].

Presently, Whole Genome Sequencing (WGS) as a high-throughput technique is gaining increasing traction in uncovering the comprehensive genome-wide diversity of *M. hyopneumoniae*. However, while this method is becoming more accessible and cost-effective, it also introduces new challenges to our existing knowledge. Nevertheless, alternative genotyping strategies like multiple-locus variable number tandem repeats analysis (MLVA) [15], amplified fragment length polymorphism (AFLP) [16], random amplified polymorphic DNA (RAPD), sequencing of variable numbers of tandem repeats (VNTRs) [17], PCR-random fragment length polymorphism (PCR-RFLP), and multi-locus sequencing typing (MLST) [18] continue to be valuable tools for delving into the realm of genetic diversity within *M. hyopneumoniae*.

This methodology is widely embraced and implemented with regard to other pathogens, aiming to establish connections between mutations in genes, including non-coding regions, at a genome-wide level and their resistance to antimicrobials. This approach facilitates a more profound comprehension of how the diminishment of bacterial fitness under the influence of antimicrobial pressure can impact the evolutionary trajectory of bacteria, steering them either toward developing resistance or remaining susceptible to the same antimicrobials [19].

While a few reports have presented the determination of MIC (Minimum Inhibitory Concentration) values for *M. hyopneumoniae* isolates, some have delved further by amplifying portions of the rRNA and/or tRNA followed by sequencing. Unfortunately, whole-genome sequencing has been somewhat overlooked in such studies by researchers [16,17,18]. Two primary challenges impede these studies. Firstly, the fastidious nature of *M. hyopneumoniae* and the labor-intensive techniques required for isolating and preserving these bacteria prove to be time-consuming, demanding well-experienced laboratory personnel. Secondly, the concern regarding AMR in *M. hyopneumoniae* remains relatively muted. Despite the growing body of research in recent years highlighting the emergence of resistant isolates to various antimicrobials across diverse geographical regions, the most effective strategy to prevent the rise in these resistant strains lies in simultaneously identifying both genotypic traits and phenotypic attributes for the same isolate. This approach leverages the capabilities of modern bioinformatics resource centers, which utilize entire genomic datasets as raw material, underscoring the truth of the adage, “An ounce of prevention is worth a pound of cure”.

### 3.2. Bioinformatic Tools and Available Genomes

Established databases serve as repositories that systematically gather and arrange data concerning AMR, primarily structured around gene ontology. These databases encompass a compilation of genes and mechanisms pertinent to AMR. Notably, within this realm, the Comprehensive Antibiotic Resistance Databases, namely CARD, and the National Databases of Antibiotic Resistance Organisms (NDARO) are highly esteemed. These repositories curate annotated reference sequences for genes that exhibit AMR attributes. Users have the capability to cross-reference their sequences against these gene ontology frameworks, facilitating precise identification and annotation of specific AMR determinants within their targeted pathogens [20].

However, the incorporation of an ever-growing multitude of AMR sequence variations into distinct functions of specific proteins engenders a degree of ambiguity in the results of these databases’ BLAST matches. This challenge necessitates augmentation through upstream bioinformatic resources, which can better manage the incorporation of these rapidly evolving AMR datasets. This enhancement is crucial for predicting associated AMR features within sequenced genomes. The Pathosystems Resource Integration Center, known as PATRIC, stands as one of the bioinformatic resource hubs endowed by the National Institute of Allergy and Infectious Diseases.

Leveraging machine learning algorithms, PATRIC furnishes classifier tools that facilitate the anticipation of AMR phenotypes and the genomic attributes aligned with these same AMR profiles.

The Comprehensive Antimicrobial Resistance Database, or CARD, offers predictions of mutations in the 23S rRNA region that confer resistance to Macrolides, Clindamycin, and Spectinomycin. Conversely, PATRIC takes a different approach by anticipating a multitude of genes associated with antimicrobial resistance (Table 1). These include EF-G, EF-Tu, gyrA, gyrB, Iso-tRNA, rpoB, rpoC, S10p, S12p, and an extensive range of proteins across a majority of the available genomes for *M. hyopneumoniae* [19].

Specifically, the rpoB and rpoC genes encode for RNA polymerase, while EF-G and EF-Tu are responsible for catalyzing the binding of an aminoacyl-tRNA to the ribosome. Moreover, these genes serve diverse moonlighting functions. S10P and S12P represent small ribosomal coding genes. In summary, PATRIC’s predictions encompass a cluster of 7 to 12 genes, many of which undergo significant changes that impact ribosomal formation and DNA synthesis, while CARD predicts conservatively between no gene to 3 AMR genes in publicly available genomes of *M. hyopneumoniae* (Table 1). Such differences have already been reported by other researchers [21,22].

## 4. In Vitro Antimicrobial Susceptibility Testing

In order to assess the potential efficacy of antimicrobials against *M. hyopneumoniae* within a living organism, it is necessary to gauge their minimum inhibitory concentration (MIC) and minimum bactericidal concentration (MBC) through in vitro measurements.

### 4.1. Minimum Inhibitory Concentration (MIC)

The MIC value represents the minimum concentration of an antimicrobial agent required to stop the growth of *M. hyopneumoniae*, whereas the reference strain is still able to grow in the identical medium without the addition of the antimicrobial agent [23]. This MIC determination offers insights into both the level of susceptibility and resistance to a specific antimicrobial.

*M. hyopneumoniae* is a slow-growing organism, which, by its very nature, causes MIC as a rather long process. Consequently, the pharmacodynamics of a particular antimicrobial might diverge from the MIC outcomes. It is wise to consider MIC results with caution, bearing in mind that such tests may sometimes fail to provide a proper prediction of the effectiveness of the antimicrobial treatment in vivo [24]. Prolonged MIC experiments and shifts in pH can potentially compromise the stability of antimicrobial agents [25].

Furthermore, a groundbreaking study conducted by Raymond et al. (2018) revealed that *M. hyopneumoniae* has the capability to reside intracellularly within an in vitro model of porcine kidney cell culture. The ability to re-isolate the bacteria after treatment with gentamicin prompts critical inquiries regarding the reliability of MIC data when applied to in vivo situations [26]. Notably, the binding of plasma proteins to antimicrobials plays a significant role, leading to potential discrepancies in MIC results between serum-free media, serum-rich media, and bodily fluids [27].

*M. hyopneumoniae* requires very stringent conditions of growth, which may take a very long time for culture; thus, the commonly used disk diffusion method for assessing bacterial susceptibility is not suitable for this organism. While some guidelines and recommendations on technical aspects of MIC testing have been published, a standardized procedure that addresses clinical breakpoints for diverse antimicrobial agents and introduces a reference strain with known MIC values for quality control is urgently needed. Additionally, MIC experiments for *M. hyopneumoniae* have employed varied methods and media, and results have been presented in differing terms such as initial MIC versus final MIC [28]. To account for the stability of the initial MIC, the lowest antimicrobial concentration is reassessed 7 to 14 days later and assigned as the final MIC. As a result, achieving consistent and correlatable MIC results among different studies poses challenges.

While it is recommended to consider the initial MIC for interpreting results of antimicrobial susceptibility tests, Felde and colleagues demonstrated that mutations in the parC gene of *M. hyopneumoniae* field isolates had negligible effects on the initial MIC values for fluoroquinolones. However, these same mutants exhibited a twelve-fold increase in final MIC values [29]. The Clinical and Laboratory Standards Institute (CLSI) [30] introduced a standardized procedure in 2011 for assessing the susceptibility of human *Mycoplasma* strains against antimicrobial agents, but no such method exists for animal *Mycoplasma* strains, especially *Mycoplasma hyopneumoniae*. The International Research Program on Comparative *Mycoplasmology* (IRPCM) advises researchers to align their MIC procedures with the guidelines published by Hannan in 2000 to ensure compatibility and comparability [30].

Clinically interpreting antimicrobial susceptibility results based on MIC involves assigning clinical breakpoint values to categorize bacterial strains as resistant or susceptible. Although such values have not been established for *M. hyopneumoniae*, previously published studies have been informally used for clinical breakpoint values [31,32]. However, using MIC values to assess antimicrobial resistance does not reveal the underlying resistance mechanism in a given strain [33]. To delve into mechanisms conferring resistance, simultaneous assessment of the genotype and phenotype features of the strain is necessary.

Major institutions like the FDA, CLSI, and EUCAST (European Committee on Antimicrobial Susceptibility Testing) publish clinical breakpoint values for various organisms. A clinical breakpoint is established by assigning an epidemiological cut-off value (ECOFF) based on the highest MIC typical for wild-type strains. The clinical breakpoint is then determined by analyzing the distribution of MIC values among wild-type strains [34]. A considerable gap between the MIC value and ECOFF indicates greater susceptibility to the antimicrobial, reducing the likelihood of developing resistant subpopulations. Conversely, antimicrobials with MIC values close to the breakpoint (MIC90) for 90% of strains could be less effective in therapy, as they are prone to selecting antimicrobial-resistant strains. It is important to note that established clinical MIC breakpoint values can evolve as microorganisms adapt in response to varying geographical conditions and dosing protocols [24].

Reports indicate that the re-isolation of *M. hyopneumoniae* and the reappearance of lung lesions are signs of antimicrobial failure and potential antimicrobial resistance [6,29,35,36,37,38,39,40,41]. Apart from AMR, three other phenomena contribute to antimicrobial therapy inefficacy: heterogeneous antibiotic resistance (heteroresistance) [42], tolerance [43], and persistence [44]. Whereas a small fraction of microorganisms, often referred to as heteroresistants, can survive and thrive at otherwise optimal antimicrobial concentrations, most are susceptible [42]. Tolerance within a population of a strain denotes the ability to survive without growth in a high antimicrobial concentration, even in the absence of resistance to the antimicrobial. Tolerance may arise from genotypic factors, involving mutations that enable microorganisms to evade the bactericidal activity of the antimicrobial agent [43]. Persistence represents a bacterial mechanism that leads to the failure of antimicrobial treatment, although the strain is susceptible to the antimicrobial agent. This phenomenon suggests that only a small bacterial subpopulation (≤1%) survives, remaining dormant by reducing metabolism, altering transcription regulation, and decreasing energy production in the presence of antimicrobial agents [44,45].

To fully realize the utility of Minimum Inhibitory Concentration (MIC) as a useful tool to optimize antimicrobial therapy, it is essential that MIC results be analyzed in concert with pharmacokinetic analysis of the same antimicrobial agent. Pharmacokinetic/Pharmacodynamic (PK/PD) methodology is commonly employed to evaluate the efficacy of antimicrobial therapy, often without considering the potential for resistance emergence. However, when coupled with in vitro growth models, PK/PD methods can be employed to explore the emergence of antimicrobial resistance mechanisms over time. In comparison to in vivo infection models, utilizing in vitro kinetic models is not only ethical but also more efficient in terms of time and cost [46].

One of the recent novel approaches was carried out by Xirui Xia et al., who applied an in vitro PK model of fitness cost for evaluating the efficiency of the antimicrobial agent tulathromycin against *M. hyopneumoniae* and the emergence of resistant strains. Applying different parameters of the dynamic model, such as AUC (antibiotic area under the curve)/MIC which characterizes the time- and concentration-dependent nature of antimicrobials, they showed that the resistant bacterial strain completely replaced the sensitive bacteria within 72 h. Remarkably, tulathromycin acted as a growth promoter, resulting in a significant increase in the bacterial population to 7 Log 10 cfu/mL after seven days of continuous culture [47,48].

Another PK/PD model study showed that multiple fractionated dose administrations of Tiamulin were bactericidal against *M. hyopneumoniae*, while the single-dose regimen was only bacteriostatic. Adjusting the antimicrobial concentration according to the %T > MIC (~3–23%) determinants of the PK/PD model led to the emergence of resistant strains of *M. hyopneumoniae* dominating the sensitive strains within 48 h. This study identified a mutation in domain V of 23S rRNA through DNA sequencing as responsible for the emergence of resistant strains [48].

### 4.2. Minimum Bactericidal Concentration or MBC

Antimicrobial agents are commonly classified as either bacteriostatic or bactericidal. The Minimum Bactericidal Concentration (MBC) is defined as the lowest concentration of an antimicrobial agent that can eliminate over 99.9% of the *M. hyopneumoniae* population within a specified timeframe, following the same methodology as the Minimum Inhibitory Concentration (MIC). When the MBC/MIC ratio is small (˂4–6), a drug is considered bactericidal, and the results of these tests often closely align with the final MIC. Achieving a concentration that can eradicate 99.9% of viable bacteria at the infection site is feasible in such cases. Conversely, if the MBC/MIC ratio is large (>6), it might be challenging to attain the same level of potency to eliminate 99.9% of bacteria, and the agent is classified as bacteriostatic [29,30].

Nevertheless, for numerous antimicrobial agents, the distinction between bactericidal and bacteriostatic effects is not absolute. The killing efficacy depends on factors like the bacterial species under investigation, the methodology employed [30], and the attainable concentration of the antimicrobial agent at the infection site. To our current knowledge, no PK/PD model or clinical breakpoints have been established based on MBC. The extension of MBC results to practical application against *M. hyopneumoniae* infections still requires further clarification. Researchers have employed various methods in MBC investigations, and in certain instances, the results can be contradictory.

While Hannan’s guideline from 2000 initially recommended a starting inoculum of 10^3^ to 10^5^ colony-forming units per milliliter (ccu/mL) or colony-forming units per plate (cfu/plate), the range still requires further refinement, as MIC values for different antimicrobial agents against *M. hyopneumoniae* continue to exhibit overlaps with higher and/or lower initial bacterial counts [30]. The Clinical and Laboratory Standards Institute (CLSI) suggests employing an inoculum of 10^4^ ccu/mL or cfu/plate to mitigate such discrepancies. The guideline mentioned above underscores that the logarithmic growth phase of *M. hyopneumoniae* inoculum has minimal impact on MIC results compared to the lag phase that might occur when recently thawed cultures are used in MIC studies [29]. However, this guideline is not universally regarded as a definitive method, and researchers still make minor and major adjustments to it [49,50]. In the unusual study, Assuncao and colleagues incorporated flow cytometry alongside the guideline to examine MIC values of various *M. hyopneumoniae* strains against nine antimicrobials. Notably, they employed an inoculum of 10^6^ ccu/mL of *M. hyopneumoniae* in the logarithmic growth phase, deviating from the usual approach [51].

## 5. Molecular Mechanisms of Antimicrobial Resistance

*M. hyopneumoniae* has undergone natural evolution, leading to alterations in functional regions of the ribosome [27,50,51], topoisomerase II, topoisomerase IV [28,38,41], and even in the expression of efflux transporters [47], all of which enable the bacterium to circumvent the cytotoxic effects of antimicrobials. Additionally, the phenomenon of biofilm formation has recently been documented in antimicrobial-resistant *M. hyopneumoniae* [6]. Biofilms are structured communities of bacteria embedded in an extracellular matrix formed by proteins, polysaccharides, and nucleic acids. In *M. hyopneumoniae*, biofilm formation helps the bacteria adhere to host tissues and protects them from external threats, such as the host immune system and antibiotics [6]. *M. hyopneumoniae* expresses several adhesin proteins, such as P97, P102, and P146, that facilitate adhesion to host respiratory epithelium [26]. Biofilms provide a physical barrier that limits the penetration of antimicrobial agents, leading to decreased antibiotic efficacy. Within biofilms, bacterial cells often exhibit altered metabolic states. For example, some bacterial cells may enter a dormant or slow-growing phase, which reduces their susceptibility to antibiotics that target actively growing bacteria [6,52]. Table 2 summarizes the known resistance mechanisms against antimicrobial agents.

### 5.1. Macrolides (rRNA)

The macrolides interact with the upper pocket of the 50S ribosomal subunit tunnel, which is largely formed by nucleotides 2058 and 2059 (*Escherichia coli* numbering) in domain V of the 23S rRNA at the entrance of the tunnel. Other neighboring nucleotides also closely engage with the binding site. Hydrogen bond formation between the amino sugar of macrolides and nucleotides 2058 and possibly 2059 positions the antimicrobials precisely. Interestingly, it has been considered that adenine at nucleotide position 2059 is highly conserved in all three life domains, whereas guanine and adenine occupy position 2058 in archaea, eukaryotes, and bacteria, respectively. The nucleotide adenine at position 2062 plays a pivotal role in the antimicrobial mechanism and is susceptible to developing antimicrobial resistance mechanisms. Macrolides interact with A2062 via their N6 amine, inducing changes in the orientation of this nucleotide within the ribosomal tunnel, ultimately leading to protein synthesis stalling [56,57]. Although the ribosomal protein L22 is positioned at the peptide exit tunnel, it does not directly contact macrolides; yet, it affects the efficiency of such antimicrobials. Changes in this protein cause distortion in an exit constriction loop, allowing the nascent polypeptide chain an escape route while the macrolide is still bound within the tunnel [54]. More recent findings instead suggest that L22 affects cell envelope component equilibria and, as such, modulates the efficiency of efflux pumps, affecting macrolide resistance [58,59]. Another mechanism of antimicrobial resistance involves the addition of a methyl group to functional center nucleotides, such as N-6 dimethylation of adenine at position 2058 [56].

The peptidyltransferase component primarily consists of domain V and is positioned within an RNA-lined cleft where no RNA protein is exposed. The introduction of antimicrobial resistance is achieved through the methylation of the adenine nucleotide at position 2503’s C-8, catalyzed by the Cfr methyltransferase [60,61]. Recent findings have unveiled previously undiscovered mutations (A2058T) that confer antimicrobial resistance in *M. hyopneumoniae* [53]. There is evidence that co-expression of Cfr methyltransferase with ErmB methyltransferase can result in resistance to all clinically effective antimicrobials in certain nosocomial strains. Vester et al. (2018) proposed that the prevalent occurrence of methyltransferase gene families in the ever-expanding pool of resistant bacterial isolates arises from the extensive utilization of macrolides against infections in both humans and animals, as well as their nontherapeutic application as growth promoters in livestock. Whereas the mono-methylation of A2058 confers a lower level of resistance, the dimethylation of this nucleotide confers higher resistance to these antimicrobials [61].

The G2057A mutation grants intrinsic resistance to erythromycin in *M. hyopneumoniae* [53]. This same mutation has been observed in other *mycoplasma* species, including *M. synoviae* [61] and *M. gallisepticum*. Similarly, the A2058G mutation has been identified as a contributor to macrolide resistance in various *mycoplasma* species beyond *M. hyopneumoniae,* such as *M. synoviae* [62], *M. genitalium* [63], *Mycoplasma bovis* [64], *M. agalactiae* [65], *M. gallisepticum* [66], and *M. hominis* [67]. The development of antimicrobial resistance comes at the cost of reduced fitness for the resistant strain, manifested as diminished competitive ability in the absence of antimicrobials. This fitness cost significantly influences resistance dynamics by exerting selection against resistance when bacteria grow without antimicrobial pressure.

Distinct mutations at positions 2058 and 2059 in domain V of the 23S rRNA result in varying degrees of fitness costs. Xia et al. (2022) demonstrated that mutant strains A2058T and A2059T of *M. hyopneumoniae* grew more slowly with lower relative fitness in the co-culture with susceptible strains [47]. Surprisingly, A2058G mutants displayed comparable or even higher fitness than wild-type strains, potentially allowing them to outcompete sensitive strains in an antimicrobial-free environment. Although the number of mutant strains studied was limited to only one for each of the mentioned mutations, other research on *M. gallisepticum* revealed that A2059G, G2073A, and A2058G mutations also incurred similar fitness costs [68].

The GTPase center of the 50S subunit is influenced by both domain II of 23S ribosomal RNA and protein L11. Methylation of the 2-O-ribose position of nucleotide A1067 at the GTPase center is known to result in antimicrobial resistance; however, such a modification’s existence in *M. hyopneumoniae* remains unverified to date [69].

Intriguingly, bacteria-producing antibiotics employ RNA methyltransferase to confer resistance traits, although this trait’s spread to sensitive microorganisms can lead to the proliferation of antimicrobial resistance.

### 5.2. Tetracyclines (Ribosomal Proteins)

Between helices h34 and h31 near the docking site where aminoacyl-tRNA binds to the 30S subunit, a high-occupancy tetracycline binding pocket is situated. Some newer variants of tetracyclines bind to this pocket with a distinct orientation, allowing them to engage with more nucleotides and consequently exhibit higher binding affinity. Given that *M. hyopneumoniae* possesses just three copies of rRNA genes in its genome, mutations targeted at these genes can readily induce resistance to tetracycline. In contrast, ribosomal protein genes are present as a single copy, rendering them more susceptible to mutations that lead to antimicrobial resistance [25].

Instances in *Ureaplasma* resistant strains have revealed that a 15bp insertion within the L4 ribosomal protein gene, resulting in a five-amino-acid insertion, can disrupt the peptide exit tunnel. This disruption facilitates the growth and exit of nascent polypeptides from the tunnel [70,71]. While there are no reports, to our knowledge, of mutations in tetracycline-resistant strains of *M. hyopneumoniae*, mutations have been observed in the ribosomal RNA of *M. bovis*, such as G1058A/C, A965T, A967T/C, and U1199C. Moreover, the presence of ribosomal protection proteins like TetM and TetO, which convey resistance to tetracyclines through mobile genetic elements or transposons, confers antimicrobial resistance in certain *Mycoplasma* and *Ureaplasma* species [64,72].

Although documentation exists regarding tetracycline resistance in *M. hyopneumoniae*, there is currently no report of mutations in the 30S ribosomal protein S10 [70,73] and S3. Notably, such mutations are prevalent in other bacterial classes [74].

### 5.3. Fluoroquinolones (Topoisomerase)

Fluoroquinolone resistance has been observed in *M. hyopneumoniae*, and several mutations have been associated with elevated MIC values [29,55]. Specific chromosomal mutations in the quinolone resistance-determining regions (QRDRs) of gyrA and gyrB genes encoding topoisomerase II, as well as parC and parE genes encoding topoisomerase IV, are linked to fluoroquinolone resistance. These alterations confer resistance to both type II topoisomerases, DNA gyrase (the primary target of quinolones), and topoisomerase IV by modifying the bicyclic skeleton of 4-quinolone through the introduction of fluorine at position C6 and a significant ring substitute at C7 [75]. This structural adaptation enables them to impede these enzymes’ functions simultaneously. While mechanisms such as efflux pump modifications, alterations in drug uptake, and changes in protein interactions have been detailed as contributors to fluoroquinolone resistance, these mechanisms have yet to be reported in *M. hyopneumoniae*.

Fluoroquinolones operate by noncovalent interaction with the cleavage-ligation active site of topoisomerase and insert at the enzyme-DNA interface at the cleaved scissile bonds. By binding to each scissile bond on both DNA strands, these drugs obstruct the ligation process during cell division. Binding to GyrA, GyrB, ParC, and ParE leads to stalling of replication/transcription complexes at the collision site, resulting in permanent chromosomal breaks. In response, the SOS response and other DNA repair pathways attempt to salvage the bacterium. If the accumulation of chromosomal breaks surpasses the repair capacity, the microorganism succumbs. This is the fundamental basis for fluoroquinolones’ bactericidal action. Additionally, quinolones hinder the overall catalytic activity of gyrase and topoisomerase IV by inhibiting DNA ligation. These dual mechanisms collectively disrupt nucleic acid processes, culminating in the bactericidal effect of these antimicrobials [76].

Functional investigations have demonstrated that despite the spatial separation between C3/C4 of fluoroquinolone molecules and serine residues at positions 83 and 87 of GyrA and ParC, a water-ion metal bridge mediates the interaction between fluoroquinolones and the enzymes. These conserved serine residues across bacterial species have been proposed as a natural resistance mechanism that safeguards bacteria against naturally occurring antimicrobials. Furthermore, mutation of either residue significantly reduces the affinity of gyrase or topoisomerase IV for quinolones, and mutation of both residues abolishes the capacity of clinically relevant fluoroquinolones to stabilize cleavage complexes, thus imparting higher resistance levels [77]. Felde et al. (2018) reported that concurrent substitutions in ParC (Glu87Gly) and GyrA (Ser80Tyr) result in increased MIC values in *M. hyopneumoniae* field isolates [29].

Other resistance mechanisms include plasmids that carry efflux pump genes which reduce intracellular fluoroquinolone concentrations, the protective Qnr proteins that shield topoisomerase-DNA complexes from fluoroquinolones by reducing the affinity between topoisomerase and DNA, overexpression of chromosome-encoded efflux pumps that reduce intracellular fluoroquinolone concentrations, and the AAC(6′)-Ib-cr enzyme that acetylates the free nitrogen on C7 of fluoroquinolones [78]. However, none of these mechanisms have been reported in *M. hyopneumoniae* thus far.

Le Carrou demonstrated that three substitutions at key positions (Ser80Phe, Asp84Asn, and Ala116Glu) in ParC are linked to a 16-fold increase in Marbofloxacin MIC values of *M. hyopneumoniae* reisolated from pigs after experimental infection [38]. The assumption of a persister subpopulation that remains non-dividing or slow-dividing, along with active efflux pumps, serves as an explanation for the reisolation of resistant strains without mutations in ParC. Double substitutions in ParC and GyrA lead to higher MIC values for *M. hyopneumoniae* against fluoroquinolones compared to single amino acid substitutions in the subunits of topoisomerases II and IV [35,51]. In alignment with these findings, it has been demonstrated in *M. gallisepticum* that the primary target of fluoroquinolones is DNA gyrase. The sequential appearance of mutations in GyrA and ParE suggests a ping-pong model, wherein the initial substitution in GryA or GryB reduces fluoroquinolone affinity, followed by subsequent substitutions in ParC and ParE as secondary targets [74]. They propose that the Ser83Arg substitution in GryA QRDR acts as a steric hindrance due to the long side chain of arginine, which does not correspond to the substitutions observed in *M. hyopneumoniae* by Vicca (2007) and Felde (2018), involving glycine, valine, and alanine, all of which are aliphatic, small amino acids with nonpolar side chains at position 83 in GryA QRDR [29,55].

### 5.4. Efflux Transporters

In the presence of antimicrobial agents, bacteria orchestrate alterations in the expression levels of various transporters or efflux genes. These efflux transporters typically fall into six evolutionarily conserved families: ATP-binding cassette (ABC) superfamily, major facilitator superfamily (MFS), multidrug and toxin extrusion (MATE), small multidrug resistance (SMR), and resistance-nodulation-cell division (RND) families. Energy for efflux of antimicrobial agents is provided by ion gradients across the bacterial membrane, with the single exception of the ABC superfamily, which uses ATP as its energy source [79].

This precludes the structural assembly of RND and ABC tripartite efflux pumps, typical for Gram-negative bacteria, within the class Mollicutes due to its lack of a cell wall. Kolesnikova et al. (2020) revealed the pivotal role of MATE family efflux transporters in the emergence of ciprofloxacin-resistant strains of *M. hominis* [80]. Tatay-Duald (2021) postulated the involvement of efflux pumps in *M. agalactiae* and *M. mycoides*, which exhibit tetracycline resistance, although the precise underlying mechanisms remain undisclosed [81]. Further instances demonstrate that efflux pumps contribute to resistance against fluoroquinolones and macrolides in *M. mycoides*, *M. hominis*, and *M. pneumoniae*, respectively [78,82,83].

Recently, Xia et al. (2022) unveiled elevated expressions of efflux transporter genes associated with the construction of ABC and MATE protein families in macrolide-resistant strains of *M. hyopneumoniae* [47]. Although these strains stem from *M. hyopneumoniae* strain J (ATCC 25934) and are not field strains, this report represents a groundbreaking revelation of resistance mechanisms beyond ribosomal RNA target mutations [53,54,55] and DNA gyrase [29,38,54] genes in the evolution of antimicrobial resistance. By introducing reserpine, a recognized efflux protein blocker, the MIC levels of *M. hyopneumoniae*-resistant strains aligned with those of sensitive strains [47,48].

## 6. Future and Horizon

Despite the economic losses incurred by Enzootic Pneumonia in the pork industry, the attention given to studying antimicrobial resistance in *M. hyopneumoniae* and its underlying mechanisms does not match that of other significant animal and human pathogens within the order Mycoplasmatales. This discrepancy can be attributed to the challenging nature of this microorganism, characterized by its fastidiousness, specific nutritional requirements, time-intensive cultivation process, and labor-intensive handling. These factors restrict the number of research groups and laboratories capable of working with *M. hyopneumoniae* at a professional level. To overcome these obstacles, a paradigm shift is needed in the essential methods and techniques for isolating and cultivating *M. hyopneumoniae*. Raymond and colleagues (2018) introduced a groundbreaking approach by demonstrating the propagation of *M. hyopneumoniae* within a porcine kidney epithelial cell line. Their findings indicated that around 8% of the bacterial population could reside intracellularly, a discovery with significant implications for antimicrobial studies, especially considering that many antimicrobial agents struggle to penetrate eukaryotic cells [26].

Another innovative avenue for exploring the genetic basis of *M. hyopneumoniae’*s antimicrobial resistance emerges from the observation of horizontal chromosomal transfer between resistant strains and susceptible bacteria under the selective pressure of antimicrobials [13,84]. This mechanism, already seen in *M. agalactiae*, highlights genetic promiscuity as a potential contributor to the development of fluoroquinolone-resistant subpopulations [85]. Furthermore, *M. hyopneumoniae’*s ability to form biofilms in the respiratory system poses an additional concern that can influence its antimicrobial resistance properties. Biofilms allow for close proximity among bacteria and thereby increase the effectiveness of horizontal chromosomal transfer within them up to 700 times more than in free-living bacteria. Biofilms also serve as physical barriers, limiting the bioavailability of antimicrobial agents and hindering their access to bacterial cells embedded within the biofilm matrix [52].

In this context, Tassew and colleagues demonstrated that field isolates of *M. hyopneumoniae* confined within biofilms could survive in antimicrobial concentrations that were tenfold higher than the MIC values required to affect their planktonic counterparts [6]. As mentioned earlier, conducting in-depth genome-wide analyses of *M. hyopneumoniae* strains with predefined antimicrobial susceptibility profiles could provide insights into the hidden genetic facets of antimicrobial resistance in *M. hyopneumoniae*. By addressing these challenges and exploring these novel avenues, researchers may uncover previously uncharted dimensions of *M. hyopneumoniae’*s response to antimicrobials.

## 7. Conclusions

This paper reviews the genetic foundation of antimicrobial resistance (AMR) mechanisms in *M. hyopneumoniae*, the causative agent of Enzootic Pneumonia in swine. *M. hyopneumoniae* is a significant economic burden due to its resistance to multiple antibiotics, which complicates treatment. AMR in *M. hyopneumoniae* results from genetic mutations, efflux pump activation, and biofilm formation. Mutations in specific genes such as 23S rRNA and gyrA have been linked to resistance, particularly against macrolides and fluoroquinolones. This study emphasizes the role of selective pressure from antimicrobials in driving genetic diversity and resistance, underlining the need for a multidisciplinary approach to combat AMR. Whole Genome Sequencing (WGS) and bioinformatic tools, such as CARD and PATRIC, are critical in predicting resistance traits and understanding the evolution of *M. hyopneumoniae*. A holistic approach which combines genomic, phenotypic, and bioinformatics data is necessary to manage AMR effectively in *M. hyopneumoniae*. Moreover, though there are some differences in the predicting AMR gene between PATRIC and CARD, with CARD being more restrictive in finding the AMR gene, the main drawback to accurate prediction of AMR genes by bioinformatic tools is the lack of whole genome sequencing (WGS) data linked with the corresponding MIC data from the same isolate and the lack of standardized antimicrobial susceptibility tests to address the rising AMR challenge in swine farming.

## Figures and Tables

**Table 1:** Comparison of 24 publicly available assembled and annotated genomes of *Mycoplasma hyopneumoniae* and prediction of antimicrobial resistance genes. CARD’s conservative approach focuses on predicting well-established AMR genes with strong evidence, while PATRIC’s machine learning and comparative genomics can predict a broader range of potential AMR genes.

Strain Name	Size (mb)	Gc%	Contigs	CDs	Isolation Country	tRNA	rRNA	Hypothetical Proteins	Proteins with Functional Assignments	Prediction of Antibiotic Resistance Genes-PATRIC	Prediction of Antibiotic Resistance Genes-CARD
232	0.89	28.6	1	674	US	28	2	311	457	10	1
J	0.89	28.5	1	672	US	29	3	274	488	8	1
7448	0.92	28.5	1	686	Brazil	30	3	298	434	11	1
168	0.92	28.5	1	694	China	29	3	389	458	9	1
168-L	0.92	28.5	1	697	China	28	2	369	463	12	1
7422	0.89	28.5	1	673	Brazil	30	3	392	497	10	1
11	0.90	28.7	5	670	The Netherland	30	3	348	484	12	1
TB1	0.91	28.7	57	602	China	30	3	287	450	12	0
KM014	0.96	28.4	1	900	Republic of Korea	29	2	348		7	1
NCTC10127	0.96	28.5	5	738	Switzerland	29	3	359	481	11	0
ES-2	0.96	28.4	1	718	China	30	2	287	463	6	1
F7.2C	0.89	28.6	1	676	Belgium	30	3	319	479	9	1
MHP691	0.90	28.5	69	625	France	30	3	298	455	12	0
MHP696	0.87	28.6	64	617	France	29	3	272	456	12	0
MHP699	0.92	28.5	74	641	France	30	3	316	457	12	0
MHP694	0.87	28.6	51	600	France	30	3	284	445	12	0
MHP709	0.87	28.6	58	619	France	30	3	272	455	12	0
MHP650	0.90	28.6	82	616	France	30	3	294	464	12	2
MHP653	0.89	28.5	74	625	France	29	3	300	462	12	0
MHP682	0.90	28.6	85	635	France	30		289	426	10	2
MHP679	0.90	28.6	102	641	France	29	3	294	458	12	0
ES-2L	0.92	28.5	1	686	China	28	2	321	408	9	1
98	0.88	28.6	19	625	The Netherland	30	3	318	121	7	3
LH	0.92	28.5	1	693	China	28	2	271	286	7	1

**Table 2:** Summary of key mechanisms of antimicrobial resistance in *M. hyopneumoniae*, including genetic mutations, efflux pumps, and biofilm formation.

Gene/Phenomenon	Mutation	Associated Antimicrobial	Resistance Mechanism	Target Molecule	References
23S rRNA	A2058G	Macrolides	Alters ribosome binding site, reduces antibiotic binding	Ribosom	[53]
23S rRNA	A2059G	Macrolides and Lincosamides	Alters ribosome binding site, reduces antibiotic binding	Ribosome	[29,53]
23S rRNA	A2064G	Macrolides	Alters ribosome binding site, reduces antibiotic binding	Ribosome	[54]
gyrA	Ser83Leu	Fluoroquinolones	Alters DNA gyrase, reduces drug binding	DNA Gyrase	[29,55]
parC	Ser80Tyr	Fluoroquinolones	Alters topoisomerase IV, reduces drug binding	Topoisomerase IV	[29,38,55]
efflux pump genes	Overexpression	Various antibiotics	Increases drug efflux, reducing intracellular concentration	ABC superfamily, MATE, MFS, RND, SMR	[47]
Biofilm formation	Phenotypic adaptation	Various antibiotics	Enhances resistance by limiting antibiotic penetration and promoting horizontal gene transfer	Extracellular Polymeric Substances, Small RNAs	[6]

## Data Availability

All data are contained within this article.
